# Assessment of Health Science Students’ Knowledge of Ocular Chemical Injuries and Proper Immediate Action: A Cross-Sectional Study at King Khalid University

**DOI:** 10.7759/cureus.70428

**Published:** 2024-09-29

**Authors:** Ashwaq Y Asiri, Ali M Alamri, Abdulkareem F Alharthi, Asma M Alharbi, Abeer B Alanazi, Furqan H Alawami, Hadeel M Albalawi, Mahmoud A Alarki, Norah A Alothman, Nouf Y Alokasi, Reema H Alyami, Raida H Aldossari, Rawan N Omar, Rafeef A Hakami, Suha A Alrumaih

**Affiliations:** 1 Ophthalmology, College of Medicine, King Khalid University, Abha, SAU; 2 Medicine and Surgery, Bisha University, Bisha, SAU; 3 College of Medicine, Umm Al-Qura University, Makkah, SAU; 4 College of Medicine, Princess Nourah Bint Abdulrahman University, Riyadh, SAU; 5 College of Medicine, Tabuk University, Tabuk, SAU; 6 Surgery, Prince Saud Bin Jalawi Hospital, Al-Mubarraz, SAU; 7 College of Medicine, King Khalid University, Abha, SAU; 8 College of Pharmacy, King Khalid University, Abha, SAU; 9 College of Medicine, King Faisal University, Al-Hofuf, SAU; 10 College of Medicine, Jazan University, Jazan, SAU; 11 College of Medicine, Imam Muhammad Ibn Saud Islamic University, Riyadh, SAU

**Keywords:** chemical injury, eye injury, knowledge and awareness, ocular injury, saudi arabia, students

## Abstract

Introduction

Chemical injuries are considered to be one of the true ocular emergencies that require years of visual rehabilitation in addition to immediate medical intervention to maintain eyesight. Thus, it is imperative that medical professionals and the general public are both aware of the damage and how to treat it.

Objective

The current study's objectives were to determine what influences students' understanding of ocular first aid in trauma scenarios and to evaluate the degree of knowledge among health university students at King Khalid University.

Methodology

Between April 25, 2024, and June 25, 2024, King Khalid University of Saudi Arabia hosted a descriptive cross-sectional study aimed at medical students. Following a thorough examination of the literature and consultation with an expert, the study researchers created an online questionnaire to gather the data. Students' academic and demographic information, their source of information, and their understanding of ocular chemical damage were all included in the final questionnaire. Using social media sites, the completed questionnaire was posted online until no further responses could be found.

Results

A total of 358 health science students completed the study survey; their ages ranged from 18 to 30 years, with a mean age of 23.5±2.4 years. A total of 55 (15.4%) students were in their pre-clinical years, 199 (55.6%) were in their clinical years, and 104 (29.1%) were medical interns. A total of 152 (42.5%) students had an overall good knowledge of ocular chemical injuries, while most of them (57.5%; 206) had an overall poor knowledge level. As for students' sources of information, the most reported were their study curricula (46.9%), internet (22.4%), doctors (11.8%), family (9.8%), and friends (8.7%), while 1.2% had no specific source. Males and being in the clinical years were significantly associated with high knowledge (p<0.05).

Conclusion

According to the study, male students in clinical study years had the greatest level of understanding regarding eye chemical injury and related first aid, while health science students had the least amount of information overall.

## Introduction

Any damage to the structure of the eye is referred to as ocular trauma (eye injuries) [1]. Nonetheless, this classification is based on three distinct types of injuries: chemical, mechanical, and viral ocular traumas [2]. The Birmingham Eye Trauma Terminology divides ocular trauma into two primary categories: open-globe injuries and closed-globe injuries. Rupture and globe laceration are the two types of open-globe injuries [3]. Penetration, perforation, and intraocular foreign materials are the three categories of lacerations [4]. This type of closed-globe injury is similar to contusion and lamellar laceration [5].

Eye injuries require very careful management since they might become serious problems if left untreated [6]. Ocular first aid is to shield the cornea from scratches and other harm [7]. Because of this, being knowledgeable about ocular first aid is essential to reacting to situations quickly. Therefore, even before a qualified medical professional arrives to give specialized care, injuries can be recognized and efficiently handled to preserve vision or provide the best possible outcome with mild degrees of impairment [8]. For the best potential visual outcome, it is imperative to identify eye injuries and take necessary action as soon as feasible [9].

Of all ocular traumas, chemical damage to the eye accounts for 11.5% to 22.1% [10]. Men make up about two-thirds of these injuries, and children between the ages of one and two years old are the most vulnerable. Industrial accidents account for the great majority of injuries that happen at work. A small percentage of injuries happen at home or as a result of an assault. Alkali compounds occur more frequently than acid injuries and are more typically encountered in building materials and cleaning agents [11]. The depth and scope of the injury should be determined by a physical examination. In particular, it is important to record the extent of involvement of the cornea, conjunctiva, and limba since this information can be utilized to forecast the final visual result [12].

Across various regions, studies on awareness of ocular chemical injuries reveal differing levels of knowledge and first-response actions. In a Saudi community study by Seraj et al., 78.5% of respondents correctly identified washing the eye with water as the first step in treating a chemical eye injury [13]. About 69% of participants in the Al-Qassim region demonstrated a fairly good level of awareness regarding ocular chemical injuries, with a statistically significant relationship between age and awareness (p<0.05) [14]. Another study in Jazan found that while 95.1% of respondents recognized the potential for ocular complications, the average knowledge score was relatively low at 7.70 out of 16, with only 59.1% identifying eye irrigation with a large amount of water as the correct initial action [15].

The current study aimed to assess medical students at King Khalid University (KKU) about their understanding of chemical ocular injuries and first aid. Since they will eventually become doctors, medical students should comprehend the tremendous implications that eye impairment can have on patients' vision. Therefore, patients should be immediately informed of the appropriate course of action to follow in the event of ocular trauma.

## Materials and methods

Study design

A cross-sectional design was employed in this study, which took place between April and June of 2024. It included all of the adult population of Saudi Arabia. Its main goals were to determine the factors influencing students' knowledge of ocular first aid in trauma cases and to evaluate the degree of knowledge among King Khalid University health science students.

Inclusion and exclusion criteria

The adult population, both male and female, who volunteered to engage in the study as health science university students at King Khalid University between the ages of 18 and 30 years met the inclusion criteria. People who did not reside in the Aseer region or who were unwilling to engage in the study were excluded.

Sampling technique

Convenient sampling was the sample method employed in this investigation. Based on their availability and willingness to participate during the data collecting period, respondents were enrolled in the study. Respondents were asked to indicate their intention to participate at the beginning of the study. While convenience sampling allowed for practical and timely recruitment, it may have introduced bias and limited the generalizability of the results, as noted in the discussion. Additionally, the use of social media for distributing the survey, though efficient, may have excluded students who are less active on these platforms, further contributing to potential selection bias. This limitation is acknowledged and justified due to the constraints of time and accessibility during the data collection process.

Sample size

The following formula was used: n = Z^2^p (1 − p)/d^2^, where n is the sample size, Z is the 95% confidence interval's critical value, 50% is the predefined proportion, and d is the 6% margin of error. We chose to employ a somewhat larger sample size of 358 respondents in order to improve the reliability of the results, even though the least acceptable sample size determined was 267.

Data collection tools and procedures

Drawing on pertinent literature, the researchers created a questionnaire that was in line with the goals and objectives of the study. Ten participants participated in the questionnaire's pilot study to evaluate its viability, readability, and comprehension. The primary study did not include the respondents who took part in the pilot project. After making modifications in response to input from the pilot, the survey was made available online to participants through groups on Telegram and WhatsApp. It included information on demographics and a section on first aid for eye injuries (see Tables 4-5 in Appendices). The knowledge in this study was assessed using a 13-item questionnaire. Each correct answer was awarded one point. Participants who scored above 7.8 points (60% of the total score) were classified as having good knowledge, while those who scored below 7.8 points (<60%) were classified as having poor knowledge.

Data analysis

The data underwent a comprehensive data cleaning process after the data collection phase was finished. The process of data cleaning involved a number of activities, including finding and eliminating duplicate entries, looking for anomalies, and filling in any gaps in the data. The information was then coded and imported into the IBM SPSS software, version 27.0 (IBM Corp., Armonk, USA). The chi-square test was utilized to ascertain the correlation between categorical variables, with a predefined significance level set at p<0.05. Categorical variables were reported as counts and frequencies. The goal of this thorough analytical method was to identify patterns in the dataset and extract insightful information.

Ethical considerations

Ethical clearance was obtained from the Abha Ethics Committee, reference number ECM#2024-1404, before data collection. The data collected adhered to anonymity by excluding personal identifiers, preventing any linkage between individual identities and study outcomes. The study respondents were informed about the study's purpose, procedures, associated risks, and potential benefits before consenting voluntarily, free from coercion.

## Results

A total of 358 health science students, with ages ranging from 18 to 30, filled out the study survey; their mean age was 23.5±2.4 years. There were 317 (88.5%) single people and exactly 286 (79.9%) females. A total of 55 students (15.4%) were pre-clinical students, 199 students (55.6%) were clinical students, and 104 students (29.1%) were medical interns. Around 103 people (28.8%) earned less than 5000 SR per month, whereas 161 people (45%) earned more than 10,000 SR per month. A total of 141 students, or 39.4%, reported having a family history of ocular injury (Table 1).

**Table 1:** Sociodemographic information of the participants Sociodemographic information presented in frequencies (N) and percentages (%).

Personal data	N	%
Age in years		
18-22	119	33.2%
23-24	138	38.5%
25-30	101	28.2%
Mean±SD	23.5±2.4	
Gender		
Male	72	20.1%
Female	286	79.9%
Marital status		
Single	317	88.5%
Married	41	11.5%
Academic year		
2^nd^ year	31	8.7%
3^rd^ year	24	6.7%
4^th^ year	65	18.2%
5^th^ year	84	23.5%
6^th^ year	50	14.0%
Medical intern	104	29.1%
Academic phase		
Pre-clinical	55	15.4%
Clinical	199	55.6%
Medical intern	104	29.1%
Income		
<5000 SR	103	28.8%
5000-10000 SR	94	26.3%
>10000 SR	161	45.0%
Do you or your acquaintances have any history of eye Injury?		
Yes	141	39.4%
No	217	60.6%

Students studying health science were aware of proper quick action and chemical harm to the eyes (Table 2). A total of 84.9% of the study participants stated that chemical injuries can result in complications for the eyes; 79.1% agreed that the initial course of treatment for ocular chemical injuries depends on the nature of the injury; and 65.9% agreed that pain intensity is a good indicator of the severity of the injury. Regarding the most severe symptom of an eye injury, pupils reported redness in 28.8% of cases and white discoloration in 24.9% of cases. Of the students, 48.9% were aware that alkaline injuries are more dangerous than acidic injuries, and 53.6% stated that one should find and remove particles if they have a chemical injury. The most common response to a chemical eye damage was to rinse the affected eye thoroughly with water (34.9%), which was followed by visiting a hospital or doctor (27.1%). Additionally, 34.1% concurred that using an alkaline solution to clean wounds caused by acidic materials and 31.6% advised using an acidic solution to clean wounds caused by alkaline materials. Of those who responded, 49.7% said washing should take five to 15 minutes, while 14% said it should take 30 minutes or longer. Furthermore, 82.1% of respondents felt that cleaning their hands after handling chemicals is a good idea before contacting their eyes. Regarding safeguards against chemical harm to the eyes, 75.4% of respondents said they would wear goggles and 17% said they would wear contact lenses.

**Table 2:** Health science students' knowledge of ocular chemical injuries and proper immediate action Health science students knowledge presented in frequencies (N) and percentages (%).

Knowledge items		N	%
Chemical injury can cause ocular complications	Agree	304	84.9%
	Disagree	24	6.7%
	I don’t know	30	8.4%
What’s the first thing you should do when a chemical eye injury occurs?	Irrigation of eye with plenty of water	125	34.9%
	Go to hospital/physician	97	27.1%
	Irrigation of eye with a little water	43	12.0%
	I don’t know	36	10.1%
	Cover the eyes	33	9.2%
	Use eye drops	24	6.7%
Initial management of ocular chemical injury varies according to the substance of ocular injury	Agree	283	79.1%
	Disagree	28	7.8%
	I don’t know	47	13.1%
Severity of pain indicates severity of ocular chemical injury	Agree	236	65.9%
	Disagree	60	16.8%
	I don’t know	62	17.3%
What is the most serious sign of ocular injury?	Eye redness	103	28.8%
	Eye white discoloration	89	24.9%
	Severe pain	72	20.1%
	Eyelid sticking	71	19.8%
	I don’t know	23	6.4%
Alkaline injuries are more dangerous than acidic?	Yes	175	48.9%
	No	77	21.5%
	I don’t know	106	29.6%
In case of chemical injury, you should locate and remove particles	Yes	192	53.6%
	No	76	21.2%
	I don’t know	90	25.1%
When injured with acidic material, wash with alkaline solution	Agree	122	34.1%
	Disagree	100	27.9%
	I don’t know	136	38.0%
When injured with alkaline material, wash with acid solution	Agree	113	31.6%
	Disagree	116	32.4%
	I don’t know	129	36.0%
What is the optimal duration of eye washing following ocular chemical injury?	< 5 minutes	77	21.5%
	5-15 minutes	178	49.7%
	30+ minutes	50	14.0%
	I don’t know	53	14.8%
Wearing goggles lowers the risk of ocular injury	Agree	270	75.4%
	Disagree	33	9.2%
	I don’t know	55	15.4%
Wearing contact lenses protects the eye against ocular chemical injury	Agree	61	17.0%
	Disagree	235	65.6%
	I don’t know	62	17.3%
After handling chemical materials, you should wash your hands before touching the eye	Agree	294	82.1%
	Disagree	30	8.4%
	I don’t know	34	9.5%

**Figure 1:**
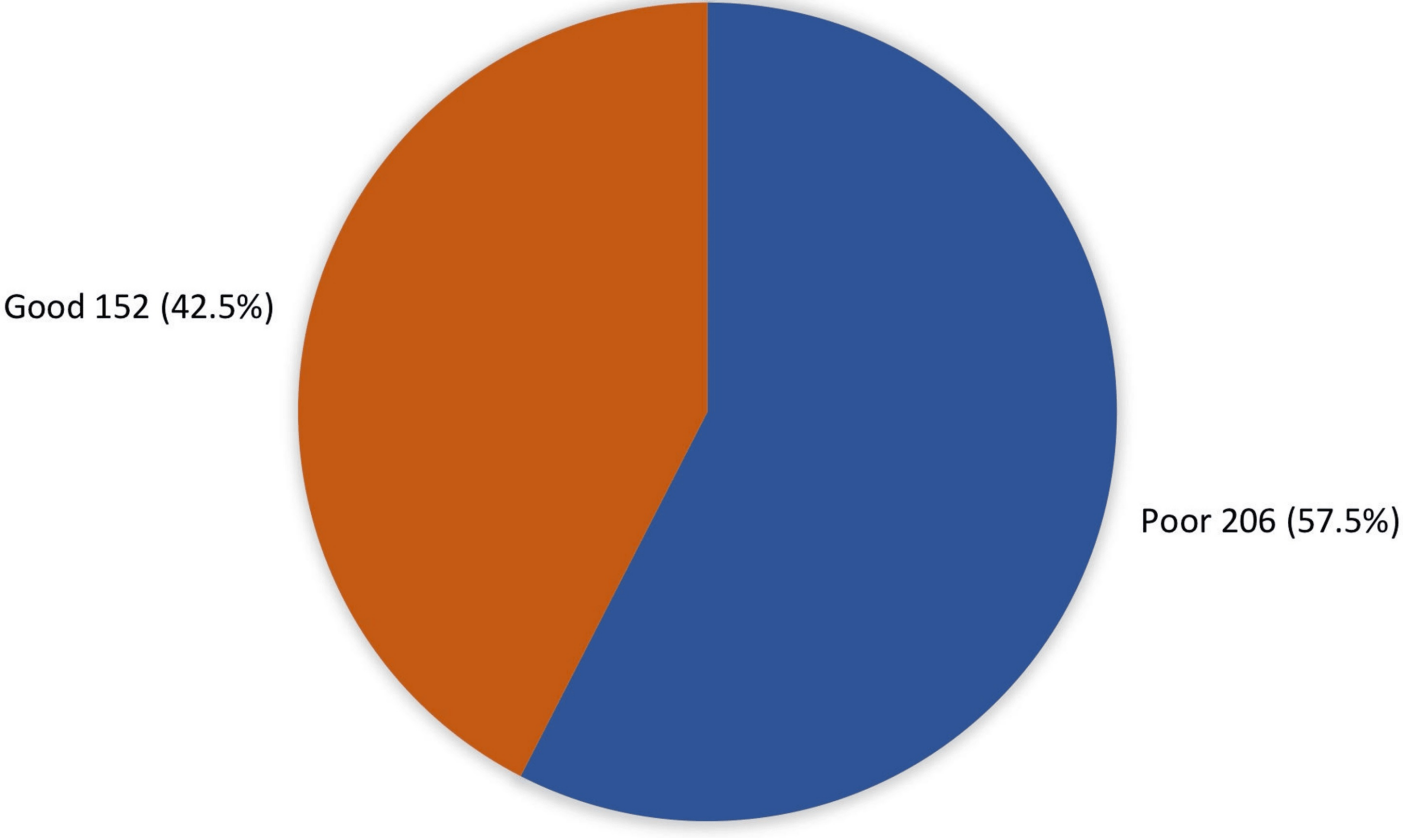
Overall knowledge level about ocular chemical injuries and proper immediate action among health science students

The overall knowledge level about ocular chemical injuries and proper immediate action among health science students is represented in Figure 1. A total of 152 (42.5%) students had an overall good knowledge of ocular chemical injuries, while most of the students (57.5%; 206) had an overall poor knowledge level.

**Figure 2:**
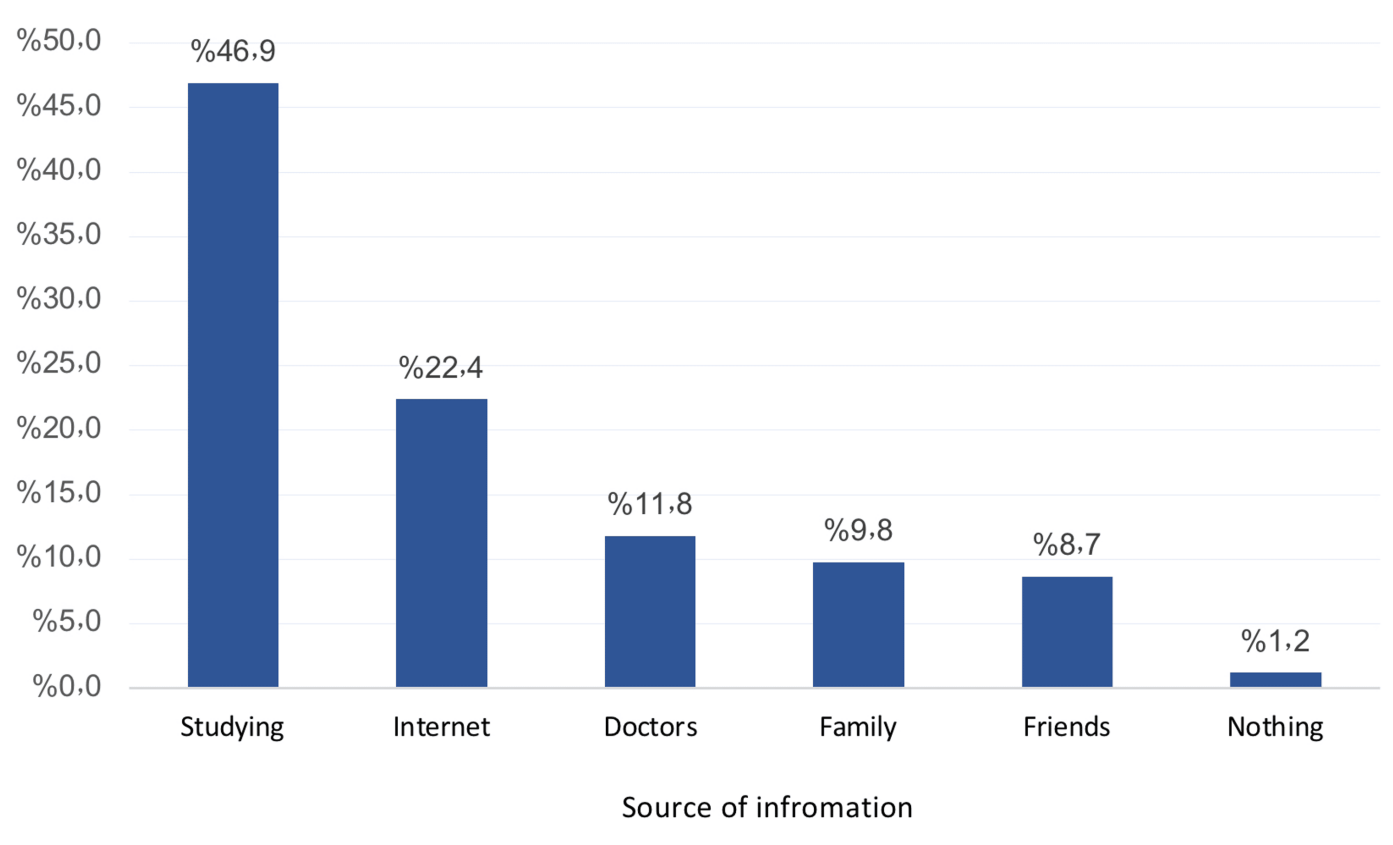
Source of students' information about ocular chemical injuries

Figure 2 depicts the source of students' information about ocular chemical injuries. The most reported were their study curricula (46.9%), internet (22.4%), doctors (11.8%), family (9.8%), and friends (8.7%), while 1.2% had no specific source.

The factors related to health science students' degree of understanding of chemical harm to the eyes are described in Table 3. In comparison to female students, 54.2% of male students possessed good overall knowledge regarding ocular chemical harm, with a statistical significance of p=0.025. Additionally, compared to 22% of married students, 45.1% of single students exhibited generally strong understanding (p=0.005). The value of p=0.001 revealed that 57.3% of low-income students had good knowledge of ocular chemical injury, compared to 37.3% of high-income students. Similarly, compared to 36.4% of students in the pre-clinical years, 47.7% of students in the clinical study years had generally strong understanding (p=0.049).

**Table 3:** Factors associated with health science student's knowledge level about ocular chemical injury *: The p-values were determined using Pearson’s chi-square test, with statistical significance set at p<0.05; ^: Exact probability test

Factors	Overall knowledge level				p-value
	Poor		Good		
	No	%	No	%	
Age in years					0.724
18-22	65	54.6%	54	45.4%	
23-24	82	59.4%	56	40.6%	
25-30	59	58.4%	42	41.6%	
Gender					0.025*
Male	33	45.8%	39	54.2%	
Female	173	60.5%	113	39.5%	
Marital status					0.005*
Single	174	54.9%	143	45.1%	
Married	32	78.0%	9	22.0%	
Academic phase					0.049*
Pre-clinical	35	63.6%	20	36.4%	
Clinical	104	52.3%	95	47.7%	
Medical intern	67	64.4%	37	35.6%	
Income					0.001*
< 5000 SR	44	42.7%	59	57.3%	
5000-10000 SR	61	64.9%	33	35.1%	
> 10000 SR	101	62.7%	60	37.3%	
Do you or your acquaintances have any history of eye Injury?					0.261
Yes	76	53.9%	65	46.1%	
No	130	59.9%	87	40.1%	
Source of your knowledge about ocular chemical injury					0.638^
Doctors	24	58.5%	17	41.5%	
Family	25	71.4%	10	28.6%	
Friends	18	58.1%	13	41.9%	
Internet	47	59.5%	32	40.5%	
None	2	66.7%	1	33.3%	
Studying	89	53.3%	78	46.7%	

## Discussion

As far as the authors are aware, there is no information available about the knowledge and awareness of King Khalid University health science students regarding eye chemical injuries and appropriate emergency first aid. Our study aims to evaluate the level of knowledge among King Khalid University health science students and identify factors that affect knowledge regarding ocular first aid in trauma cases. Additionally, the risk factors described in other populations are similar to those of the study subject. According to the World Health Organization's Blindness Data Bank, 19 million of the 55 million eye injuries that occur each year result in blindness or visual loss [16]. Ocular trauma may happen to anyone at any age, however it is most commonly reported to have occurred in young boys. Since individuals between the ages of 22 and 44 years account for 10% of cases of bilateral blindness, these injuries may have a long-term effect on those patients' vision [17]. 

Less than half of the students had an overall strong understanding of ocular chemical damage, according to the results of the current study. About one-third of the pupils were aware of the nature of injuries and harmful materials, compared to the vast majority who were aware of the serious indicators and repercussions associated with trauma. Regarding first aid, around one-third of them mentioned rinsing the eyes with lots of water; over 50% mentioned clearing away chemical particles, but less than 3% mentioned using an appropriate eye irrigation solution. More than three-fourths of the respondents said they used goggles as a kind of protection, but very few mentioned contact lenses. The study curricula were the most frequently cited information sources, while reports also came from the internet and medical professionals. During their clinical years, male students had noticeably greater knowledge levels. This could be attributed to the male students having more hands-on experiences or been more actively involved in clinical settings, which could lead to better retention and understanding of topics related to ocular chemical injuries. Additionally, in some contexts, male students might have had more opportunities to engage with certain medical scenarios, either due to societal norms or expectations, which could have provided them with more exposure to relevant information [18].

Research revealed that only 27% of King Abdulaziz University medical students knew anything about ocular trauma and first aid [19]. This is a far lower level of knowledge than was previously thought. While sixth-year students had the highest amount of knowledge, third-year students had the highest percentage of inadequate knowledge. Additionally, the study was the most often cited information source, which is consistent with the results of the current study. A total of 240 (72.1%) medical students responded that the appropriate course of action in this kind of injury is vigorous irrigation with copious amounts of clean water. In contrast, the study showed that more than half of the students were aware that the level of danger in alkaline damage is higher than that in acidic injury.

Nonetheless, the following were the study's misconceptions: Compared to the current study students' knowledge level, 29 (8.7%) students decided to wash with an acidic solution after being damaged by an alkali, and 32 (9.6%) students chose to wash with an alkaline solution. According to Alqassim et al. [15], 40 participants (7.5%) reported that they thought acidic solutions should be administered to alkaline burns, while 25 participants (4.7%) thought the opposite, which is significantly less than the awareness that was measured in our study.

Both washing techniques carry a very significant danger of exothermic reactions and subsequent thermal damage. Higher than the level evaluated in the current study, another study conducted in Al-Quassim revealed that 69% of the participants had a reasonably high level of awareness regarding ocular chemical harm. The majority of participants (93.8%) thought that ocular chemical injury could result in ocular difficulties, and 92.2% of them named detergents and chloride as the primary materials that cause eye injuries [14]. About 48% of respondents in the Aseer region reported having a similar level of awareness of chemical eye harm [20]. Globally, Harakuni and Kumar evaluated high awareness of chemical harm to the eyes and related treatment [21].

Nevertheless, the study was marred by a few limitations, including its focus solely on King Khalid University health science students, which restricts the generalizability of the findings to other institutions or countries. This focus on a single institution means that the results may not fully reflect the broader knowledge levels or attitudes of health science students elsewhere. Secondly, the reliance on self-reported data introduced a potential bias, such as overestimation of knowledge and socially desirable responses. Additionally, the study primarily emphasizes curriculum as the main knowledge source but overlooks other influential factors like personal study habits, interests, and external clinical exposures. The study adopted cross-sectional design which prevents the establishment of causal relationships between the curriculum and observed knowledge levels. Addressing these limitations in future research could lead to more comprehensive and effective strategies to enhance ocular first aid knowledge among health science students.

## Conclusions

The current study's findings showed that health science students' understanding of ocular chemical injury and its initial treatment was lacking. There was also a reported increased knowledge of the environment, its effects, and preventative measures. During the clinical study years, male students had the highest degree of understanding. The study curriculum served as the main source of information for medical students, emphasizing the importance of increasing their exposure to ocular first aid management. Consequently, the authors suggest training students with the appropriate first aid skills that could save their vision and exposing them to more examples of ocular injuries through clinical settings and simulation sessions.

## Appendices

**Table 4:** Personal information

Question	Options
Gender	Female
	Male
Age	_______
Marital status	Married
	Single
Academic year	Second Year
	Third Year
	Fourth Year
	Fifth Year
	Sixth Year
	Internship Year
Family income per month (SR)	<5000 SR
	5000-10000 SR
	>10000 SR
Do you or your acquaintances have any history of eye injury?	No
	Yes
What is the source of your knowledge about ocular chemical injury?	Internet
	Friends
	Family
	Doctors
	Studying
	Others (specify) _______

**Table 5:** Eye injury first aid knowledge

Question	Options
Chemical injury can cause ocular complications	Agree
	Disagree
	I don’t know
What’s the first thing you should do when a chemical eye injury occurs?	I don’t know
	Go to hospital/physician
	Use eye drops
	Cover the eyes
	Irrigation of eye with a little water
	Irrigation of eye with plenty of water
	Others (specify) _______
Initial management of ocular chemical injury varies according to the substance of ocular injury	Agree
	Disagree
	I don’t know
Severity of pain indicates the severity of ocular chemical injury	Agree
	Disagree
	I don’t know
What is the most serious sign of ocular injury?	Eye redness
	Eye white discoloration
	Eyelid sticking
	Severe pain
Alkaline injuries are more dangerous than acidic injuries	Yes
	No
	I don’t know
In case of chemical injury, you should locate and remove particles	Yes
	No
	I don’t know
When injured with acidic material, wash with alkaline solution	Agree
	Disagree
	I don’t know
When injured with alkaline material, wash with acid solution	Agree
	Disagree
	I don’t know
What is the optimal duration of eye washing following ocular chemical injury?	5-15 min
	<5 min
	30 min or more
	I don’t know
Wearing goggles lowers the risk of ocular injury	Agree
	Disagree
	I don’t know
Wearing contact lenses protects the eye against ocular chemical injury	Agree
	Disagree
	I don’t know
After handling chemical materials, you should wash your hands before touching the eye	Agree
	Disagree
	I don’t know
